# Abnormal HCK/glutamine/autophagy axis promotes endometriosis development by impairing macrophage phagocytosis

**DOI:** 10.1111/cpr.13702

**Published:** 2024-07-02

**Authors:** Sha‐Ting Lei, Zhen‐Zhen Lai, Shu‐Hui Hou, Yu‐Kai Liu, Ming‐Qing Li, Dong Zhao

**Affiliations:** ^1^ Department of Obstetrics and Gynecology, Shanghai Ninth People's Hospital Shanghai Jiao Tong University School of Medicine Shanghai China; ^2^ Laboratory for Reproductive Immunology, Hospital of Obstetrics and Gynecology Fudan University Shanghai China; ^3^ Department of Gynecology, Shanghai First Maternity and Infant Hospital Tongji University School of Medicine Shanghai China; ^4^ Department of Reproductive Immunology, The International Peace Maternity and Child Health Hospital, School of Medicine Shanghai Jiao Tong University Shanghai China

## Abstract

The presence of extensive infiltrated macrophages with impaired phagocytosis is widely recognised as a significant regulator for the development of endometriosis (EMs). Nevertheless, the metabolic characteristics and the fundamental mechanism of impaired macrophage phagocytosis are yet to be clarified. Here, we observe that there is the decreased expression of haematopoietic cellular kinase (HCK) in macrophage of peritoneal fluid from EMs patients, which might be attributed to high oestrogen and hypoxia condition. Of note, HCK deficiency resulted in impaired macrophage phagocytosis, and increased number and weight of ectopic lesions in vitro and in vivo. Mechanistically, this process was mediated via regulation of glutamine metabolism, and further upregulation of macrophage autophagy in a c‐FOS/c‐JUN dependent manner. Additionally, macrophages of EMs patients displayed insufficient HCK, excessive autophagy and phagocytosis dysfunction. In therapeutic studies, supplementation with glutamine‐pre‐treated macrophage or Bafilomycin A1 (an autophagy inhibitor)‐pre‐treated macrophage leads to the induction of macrophage phagocytosis and suppression of EMs development. This observation reveals that the aberrant HCK‐glutamine‐autophagy axis results in phagocytosis obstacle of macrophage and further increase the development risk of Ems. Additionally, it offers potential therapeutic approaches to prevent EMs, especially patients with insufficient HCK and macrophage phagocytosis dysfunction.

## Graphical abstract

1. The deficiency of HCK impairs macrophage phagocytosis in EMs patients.
2. Macrophage phagocytosis induced by the HCK is dependent on glutamine level.
3. Glutamine maintains macrophage phagocytosis by suppressing the c‐FOS/c‐JUN signal pathway‐mediated autophagy.
4. Supplementation with glutamine‐pre‐treated macrophage or Bafilomycin A1‐pre‐treated macrophage induces macrophage phagocytosis and suppresses EMs development.

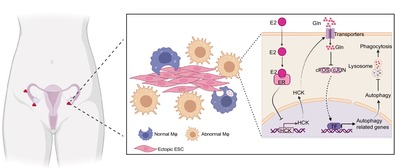

## 1 INTRODUCTION

Endometriosis (EMs) is a prevalent gynaecological condition that is dependent on oestrogen. It is a benign disease characterised by the abnormal presence of endometrial stroma and glands outside the uterus (such as ovaries, peritoneum fallopian tubes, bladder or intestines) accompanied with chronic inflammation.^1^, ^2^ EMs affects approximately 10% of reproductive‐age women globally, and manifests as chronic pelvic pain, dyspareunia and infertility.^1^, ^3^ Unfortunately, the pathogenesis of EMs still has not been figured out yet, and the clinical therapies for EMs are far from satisfactory.^2^, ^4^, ^5^

Accumulating evidence suggested that immune dysregulation had a crucial part in the onset and development of this mysterious disorder, alongside hormonal and inherent abnormalities of the endometrium, such as abnormal number and function of macrophages, abnormal differentiation and function of T‐ and B‐cells, and reduced cytotoxicity of NK cells.^6^, ^7^, ^8^, ^9^ The phagocytosis function of macrophages is compromised due to the reduced CD36 expression and heightened expression of signal regulatory protein α (SIRPα) in EMs patients, contributing to incomplete endometrial shedding, presence and survival of desquamated tissues in the peritoneal cavity.^10^, ^11^ The cellular metabolic pathways mainly include glycolysis, lipid metabolism, glutamine metabolism and oxidative phosphorylation.^12^ In macrophages, metabolic and immune pathways are strongly interconnected. Investigation indicated that glutamine metabolism plays a crucial role in proliferation and activation in macrophage.^13^ According to recent research, the process of macrophage phagocytosis can be affected by cellular metabolism (i.e., glycolysis, oxidative phosphorylation, lipid synthesis, arginine and heme).^14^, ^15^, ^16^, ^17^, ^18^ However, the metabolic mechanism underlying the impaired macrophage phagocytosis remains largely unidentified. The in‐depth understanding of macrophage phagocytosis in endometriotic milieu might shed light on the development of therapeutic targets and strategies for EMs.

Haematopoietic cellular kinase (HCK), which belongs to the cytoplasmic tyrosine kinases of the SRC family, is highly expressed in phagocytes, including macrophages, neutrophils, dendritic cells and mast cells. Investigators have demonstrated that HCK plays important roles in adhesion and migration, podosome formation, actin polymerisation and lysosome exocytosis.^19^ It was published that aberrant autophagy of endometrial stromal cells (ESCs) was involved in pathogenesis of EMs by multiple pathways, including promoting invasion of ESCs, and inducing immune dysfunction.^20^, ^21^, ^22^ Our previous studies suggested that the downregulation of HCK in endometriotic ESCs with autophagy suppression, indirectly resulted in the impaired cytotoxicity NK cell.^22^ More importantly, it has been reported that HCK^23^, ^24^ and cell autophagy^25^, ^26^, ^27^ play a crucial role in the regulation of monocyte/macrophage recruitment, activation and function. However, the role of HCK and autophagy in macrophage phagocytosis has not been addressed yet.

Hence, the objective of this research was to examine the governing mechanisms of HCK‐metabolism‐autophagy in the process of macrophage phagocytosis, and its pathogenic roles in EMs and possible approaches for intervention both in vitro and in vivo.

## 2 MATERIALS AND METHODS

### 2.1 Collection of samples from patients

The research protocol for this study has been approved by the Human Research Ethics Committee of Obstetrics and Gynecology Hospital of Fudan University (ethical No. 2019‐103‐C1), and the Medical and Ethical Committee of the Shanghai First Maternity and Infant Hospital affiliated with Tongji University (ethical No. KS1742). Each participant signed a written informed consent form. All of the EMs peritoneal fluid (PF) was obtained by laparoscopy from 30 patients with EMs (range 22 to 47 years) at the Obstetrics and Gynecology Hospital of Fudan University and Shanghai First Maternity and Infant Hospital. Control PF was collected from patients with leiomyomas undergoing hysterectomy (29 cases). None of the enrolled patients experienced any complications related to pelvic inflammatory disease, and they did not use any medications or undergo hormonal therapy within 6 months of the surgery. The samples were collected during the secretory phase of the menstrual cycle, as confirmed by histological examination.

### 2.2 Collection and preparation of PF

PF was aspirated from the *cul de sac* at the start of surgery with sterile syringe. Blood‐contaminated PF samples were excluded from the study. Lymphocyte separation medium (Solarbio Life Science, Beijing, China, P8610) was employed for isolating mononuclear cells from PF, and then subjected to density‐gradient centrifugation at 300 × *g* for 20 min at 4°C. Subsequently, the harvested cells were rinsed with ice‐cold phosphate‐buffered saline (PBS; GENOM Co., Ltd, Hangzhou, China, GNM‐20012) for further experiments.

### 2.3 Isolation and purification of PM cells

The PF cells, isolated from PF of both the control group and patients with EMS, were collected to isolate macrophages with MASC, a human/mouse CD14^+^ cell isolation kit (130‐050‐201, Miltenyi Biotec, Germany) for in vitro experiments.

### 2.4 Immunofluorescence

The PM cells isolated from PF of both the control group and patients with EMS were grown on autoclaved coverslips. Cells were immobilised on cover slips previously treated with poly‐l‐lysine (1 mg/mL, Sigma‐Aldrich), and then were fixed with 4% paraformaldehyde (PFA) for 20 min and permeabilised with 0.4% Triton X‐100 in PBS for 5 min, and blocked with 5% BSA/0.05% Triton X‐100/4% goat serum in PBS for 1 h at room temperature and incubated with the following antibodies at 4°C overnight: HCK (1:100; Thermo Fisher, PA5‐109806). Secondary antibodies used and dilution ratios were as follows: dinkey anti rabbit (1:500; Abcam, ab150075). Nuclei were stained with DAPI (D1306, Thermo Fisher Scientific) for 3 min. Confocal images were collected using 1024 × 1024 image format and 63 × optical zoom. In the absence of primary antibodies, staining of secondary antibodies (negative controls) failed to produce any significant staining.

### 2.5 Mice

*Hck* heterozygous mice were obtained from The Jackson Laboratories (Sacramento, CA, USA) and were then kept at the Shanghai Model Organisms Center, Inc. (Shanghai, Shanghai, China). For this study, we acquired female C57BL/6 mice from the esteemed Shanghai Slac Laboratory Animal CO.LTD in Shanghai, China. Prior to the commencement of the experiment, the mice were housed in the animal facility for a duration of 2 weeks. All animal procedures were conducted with the approval of the Animal Welfare Committee of Shanghai Model Organisms Center, Inc.

### 2.6 EMs mice model

An intraperitoneal EMs model was constructed. Donor mice were randomly selected from a group of female WT mice, and their uterus horns were removed and minced into fragments smaller than 1 mm^3^. The fragments were subsequently placed in sterile saline and administered to female WT mice or *Hck* ^−/−^ mice via intraperitoneal injection (the ratio of uterine to intraperitoneal injection in recipient mice was 1:2). The receptors were sacrificed after 14 days, and endometriotic lesions were collected.

### 2.7 Culturing cell lines

HESCs were acquired from the American Type Culture Collection (CRL‐4003; ATCC, Manassas, VA, USA), and maintained in DMEM/F‐12 medium containing 10% FBS (Hyclone; GE Healthcare), 100 U/mL penicillin and 100 μg/mL streptomycin (Gibco), and incubated at 37°C in a humidified atmosphere with 5% CO_2_. THP‐1 human monocytes were obtained from the ATCC (TIB‐202; Manassas, VA, USA), and cultured in Roswell Park Memorial Institute (RPMI)‐1640 medium containing 10% FBS, 100 U/mL penicillin and 100 μg/mL streptomycin (Gibco), and incubated in a humidified incubator with 5% CO_2_ at 37°C.

### 2.8 Induction of macrophages

THP‐1 cells were used to stimulate the differentiation into macrophages. THP‐1 cells, seeded in 6‐well plate (5 × 10^5^/well), were treated with phorbol 12‐myristate 13‐acetate (PMA, 100 ng/mL; Sigma‐Aldrich, 16,561–29‐8) for 24 h to induce M0 macrophages and both recombinant human (rHs) IL4 (20 ng/mL, PeproTech), and rHs‐IL13 (20 ng/mL, PeproTech) for 24 h to induce M2 macrophages.

### 2.9 Cell transfection

To downregulate the HCK gene in THP‐1 cells, recombinant lentivirus carrying siRNA targeting *HCK* (HCKi) (Genechem, Shanghai, China) or a control lentivirus containing GFP (Genechem, Shanghai, China) was used to infect THP‐1 for 10 h. After 10 h of infection, fresh complete culture medium was used for further culture. Infection efficiency was evaluated 72 h after infection using real‐time polymerase chain reaction (RT‐PCR) and western blotting.

### 2.10 In vitro co‐culture assay and phagocytosis assay

The PM cells of patients with or without Ems, or PM cells of *Hck* ^−/−^ mice were plated in six‐well plates and incubated with carboxylate‐modified microspheres (F8827, Life Technologies, USA) for 1 h at 37°C, using a macrophage to bead ratio of 1:100. After the incubation, the supernatant was discarded, and the cells were washed three times with ice‐cold PBS. The fixed cells using 4% formaldehyde were then analysed for the percentage of intracellular fluorescent beads through flow cytometry (FCM) or observed under confocal fluorescence microscopy. Phagocytosis was calculated as the percentage of CD14^+^FITC^+^ cells among CD14^+^ macrophages.

HCKi M2 macrophages or PM cells of *Hck* ^*−/−*^ mice were cultured in six‐well plates. Chicken erythrocytes (Solarbio, China) were labelled with PKH26 Red Fluorescent Cell Linker Midi Kit (MIDI26, Sigma‐Aldrich) according to the manufacturer's instructions. The cells were extensively washed and co‐cultured with macrophages for 1 h at 37°C, using a macrophage to chicken erythrocytes ratio of 1:10. HESCs labelled with PKH26 or CFSE (Cell Division Tracker Kit, 423801, Biolegend) were also used to co‐culture with macrophages for 2 h at 37°C, using a macrophage to HESCs ratio of 1:2. After co‐culture, the cells washed with PBS twice and then phagocytosis rate was examined by fluorescence microscopy. Alternatively, the cells were digested with trypsin and gathered in the centrifuge tube, and their phagocytosis rate detected by FCM.

### 2.11 In vivo phagocytosis assay

The in vivo co‐culture system was constructed by intraperitoneal injection of pre‐treated macrophages of *Hck* ^−/−^ mice or WT mice into recipient mice, once a week. Briefly, macrophages were isolated from mouse spleen and labelled with PKH26 Red Fluorescent Cell Linker Midi Kit. And then, macrophages from WT mice were treated with DMSO (0.1%; Sigma‐Aldrich, 67–68‐5), glutamine (4 mM; Sigma‐Aldrich, 1294808) for 4 h, and bafilomycin a1 (Baf, 100 μM, MedchemExpress, HY‐100558) for 1 h. After 2 weeks of co‐culture in vivo, peritoneal cells were collected from the mice and detected using FCM. Phagocytosis was calculated as the percentage of Vimentin^+^PKH26^+^ macrophages cells among donor macrophages.

### 2.12 Metabolomics testing (GC–MS analysis)

Metabolomics testing was conducted on peritoneal macrophage (PM) cells of *Hck* ^−/−^ mice (*n* = 8) or WT mice (*n* = 10). The cells were disrupted and lysed using sonication (30,000 Hz) in pre‐chilled PBS buffer. Subsequent to repeated centrifugations with 85% acetone, the supernatant was collected to obtain prepared free metabolites and proteome. The chromatography was performed using Gas chromatography–mass spectrometry (GC–MS) detection (Thermo, Ultimate 3000LC, Orbitrap Elite). The detailed experimental conditions for this analysis were described in the previous.^28^

### 2.13 Transmission electron microscopy detection

PM cells from *Hck* ^−/−^ mice (*n* = 4) or WT mice (*n* = 4) were gathered and fixed in 2.5% glutaraldehyde and post‐fixed in 1% osmium tetroxide. After dehydrating in increasing concentration of alcohol, the samples were embedded in epoxy resin (Sigma‐Aldrich, 45,345). The sections were sliced, stained with uranyl acetate and lead citrate and then examined using a Philips CM120 transmission electron microscope (Philips, Amsterdam, Netherlands). The autophagosome, a vacuole structure with double layers containing mitochondria, endoplasmic reticulum and ribosomes, was observed in the middle stage of autophagy. In the late stages of autophagy, autolysosomes with single‐layer membranes and degraded cytoplasmic components are observed. Take three fields of view for each sample, count the number of autophagosomes and take the mean as the value of the sample, followed by data statistics.

### 2.14 DAPGreen autophagy detection

M2 macrophages were plated on μ‐slide 8 well (Ibidi, 80827) and incubated overnight. The cells were rinsed with culture medium and then exposed to 250 μL of 0.1 μmol/L DAPGreen working solution (DAPGreen‐Autophagy Detection; Dojindo, D676) at 37°C for 30 min. After being washed with the culture medium twice, the cells were subjected to DMSO (0.1%, Sigma‐Aldrich, 67–68‐5), Gln‐free medium (Gibco, 21870076), or rapamycin (100 nM, MedChemExpress, HY‐10219) for 4 h or bafilomycin A1 (Baf, 100 μM, MedChemExpress, HY‐100558) for 1 h. M2 macrophages were cultured with glutamine‐free or regular growth medium with/without T5224 (10 μM, MedChemExpress, HY‐12270) for 4 h. The cells were rinsed with Hanks' buffer solution twice and then DAPGreen fluorescence was observed using confocal fluorescence microscopy. Alternatively, the cells were rinsed with PBS, treated with trypsin and centrifuged. The pellets were suspended in Hanks' buffer solution, and analysed using FCM. Confocal microscopy (Leica) was using to capture fluorescence images at an excitation wavelength of 425–475 nm and a 500–560 nm emission filter. These data were acquired using a flow cytometer (Beckman) at an excitation wavelength of 488 nm and a 500–560 nm emission filter. The experiments were conducted in triplicate.

### 2.15 Western blotting

The cells were washed in PBS, detached using a cell scraper and the centrifuged at 10,000 × *g* for 30 min at 4°C. The resulting pellet was re‐suspended in high‐efficiency cellular tissue rapid lysis buffer (RIPA; Beyotime, P0013J) with 1% phenylmethanesulfonylfluoride (PMSF; Beyotime, P1008) proteinase and 1% phosphatase inhibitors (Roche Diagnostics, 04693132001). Subsequently, the cell lysates were boiled at 95°C for 10 min, and stored at −80°C. The protein quantity was determined using the BCA protein assay kit (Beyotime, P0012). A total of 15 μg of proteins were electrophoresed in SDS‐PAGE gels (Epizyme Biotech, LK102) using a Miniprotein III system (Bio‐Rad, USA) and then transferred to PVDF membranes (Millipore, USA) for 1 h. This was followed by overnight incubation with primary antibody against ATG5 (1:1000; Cell Signaling Technology, D5F5U), ATG7 (1:1000; Abcam, ab133528), BECLIN1 (1:1000; Cell Signaling Technology, D40C5), EGR1 (1:1000; Abcam ab133695), HCK (1:1000; Cell Signaling Technology, E1ITF), LC3B (1:1000; Cell Signaling Technology, 2775), p62 (1:1000; Abcam, ab56416), c‐FOS (1:1000; Cell Signaling Technology, 9F6), c‐JUN (1:1000; Cell Signaling Technology, 60A8) and GADPH (1:1000; Cell Signaling Technology, 14C10) at 4°C. PVDF membranes were then washed three times with TBST solution (Sangon Biotech, C520009) and subsequently incubated at room temperature for 1 h in peroxidase‐conjugated goat anti‐rabbit IgG secondary antibodies (1:5000; Bioworld Technology, Co. Ltd., Louis Park, MN). Following the three washes, the membrane was subjected to chemiluminescence using Immobilon Western Chemiluminescent HRP Substrate Kit (Millipore).

### 2.16 FCM analysis

Antibodies derived from human and mouse were employed in FCM assays to quantify cellular markers. Complete information regarding all antibodies utilised can be found in Table 1. Human Trustain FcX (Biolegend, CA, USA) was specifically utilised to obstruct Fc receptors before the commencement of FCM. Following this, the cells were rinsed twice and resuspended in PBS for FCM analysis. The samples were assessed using a CytoFLEX flow cytometer (Beckman Coulter, Inc.) and data were analysed using FlowJo (version 10.07 (FlowJo LLC)).

**TABLE 1:** Antibodies for flow cytometry assays.

Antibody	Fluorescence	Manufactory	Cat No.
Anti‐human CD45	APC/Cyanine 7 (APC/Cy7)	Biolegend	368516
Anti‐human CD14	APC	Biolegend	325608
Anti‐mouse CD45	APC/Cy7	Biolegend	103116
Anti‐mouse F4/80	FITC	Biolegend	123108
Anti‐mouse CD11b	Brilliant violent (BV) 650	Biolegend	301336
Anti‐human/mouse Vimentin	Allophycocyanin (APC)	R&D	IC2105A

### 2.17 Quantitative real‐time polymerase chain reaction

The PM cells of patients with or without EMs, PM cells of WT or *Hck* ^−/−^ mice, HCKi THP‐1 cells, estradiol (E2, 0 nM, 10 nM, 100 nM) treated THP‐1 cells for 24 h, glutamine‐free RPMI‐1640 or containing glutamine (4 mM) RPMI‐1640 cultured THP‐1 cells for 4 h, glutamine (0 mM, 2 mM, 4 mM) treated NC or HCKi THP‐1 cells for 4 h.

The total RNA was isolated using TRIzol regent (Invitrogen, Carlsbad, CA, USA). Following this, the concentration and purity of RNA was assessed with a NanoDrop spectrophotometer (NanoDrop Technologies; Thermo Fisher Scientific, MA, USA). The PrimeScript RT Reagent Kit (TaKaRa Biotechnology, Co., Ltd., Dalian, China) was employed to perform reverse transcription of the total RNA into cDNA. Subsequently, qRT‐qPCR was conducted using SYBR Green PCR Master Mix (TaKaRa Biotechnology). The quantitative real‐time polymerase chain reaction primers can be found in Table 2. The expressions of the target mRNA were normalised to *GAPDH* or *Gapdh* expression. All reactions were carried out on the Applied Biosystems 7500 Real‐Time PCR System (Thermo Fisher Scientific, MA, USA.). The analysis of the test results was performed using the 2^−ΔΔCt^ method.

**TABLE 2:** Primer sequences of each gene detected in RT‐PCR.

Gene	Sequence	
*GADPH*	Forward	5′‐TGGTGAAGGTCGGTGTGAAC‐3′
	Reverse	5′‐GCTCCTGGAAGATGGTGATGG‐3′
*ATG5*	Forward	5′‐AAAGATGTGCTTCGAGATGTGT‐3′
	Reverse	5′‐CACTTTGTCAGTTACCAACGTCA‐3′
*ATG7*	Forward	5′‐CTGCCAGCTCGCTTAACATTG‐3′
	Reverse	5′‐CTTGTTGAGGAGTACAGGGTTTT‐3′
*BECN1*	Forward	5′‐GGAGCTGCCGTTATACTGTTCTGG‐3′
	Reverse	5′‐TGCCTCCTGTGTCTTCAATCTTGC‐3′
*c‐FOS*	Forward	5′‐CTTCCCAGAAGAGATGTCTGTG‐3′
	Reverse	5′‐TGGGAACAGGAAGTCATCAAAG‐3′
*c‐JUN*	Forward	5′‐CAAACCTCAGCAACTTCAACC‐3′
	Reverse	5′‐CTGGGACTCCATGTCGATG‐3′
*EGR1*	Forward	5′‐GGGCAGCGGCAGCAACAG‐3′
	Reverse	5′‐TGCGGTCAGGTGCTCGTAGG‐3′
*HCK*	Forward	5′‐CTCCAGGTCGGAGGCAATAC‐3′
	Reverse	5′‐GAGCCTGCCTCCCTGATTC‐3′
*MAP1LC3B*	Forward	5′‐CCGACTTATTCGAGAGCAGCATCC‐3′
	Reverse	5′‐GTCCGTTCACCAACAGGAAGAAGG‐3′
*SLC1A4*	Forward	5′‐TATGTGCTCAGCGACCCTTC‐3′
	Reverse	5′‐CGCTGTGGCAGTCACTAGAA‐3′
*SLC1A5*	Forward	5′‐TTGATCCTGGCTGTGGACTG‐3′
	Reverse	5′‐CTCCGTACGGTCCACGTAAT‐3′
*SLC38A1*	Forward	5′‐CCTCACAGTGCCGGTGTTAT‐3′
	Reverse	5′‐TGCAGGTAACCACGGTATGAC‐3′
*SLC38A2*	Forward	5′‐GCTGTGACCCTGACAGTACC‐3′
	Reverse	5′‐ACTATGACGCCACCAACTGA‐3′
*SLC38A5*	Forward	5′‐CTGAGAGGAGCAGGATGGAAC‐3′
	Reverse	5′‐CTCACGTTCTTGCCTGTAGC‐3′
*SLC38A7*	Forward	5′‐ATCATCATTGGCGACCAGCA‐3′
	Reverse	5′‐ATGGTGAACTTGCGGTCTGT‐3′
*TNF‐α*	Forward	5′‐TCCCCAGGGACCTCTCTCTAA‐3′
	Reverse	5′‐AGCTTGAGGGTTTGCTACAACAT‐3′
*Gadph*	Forward	5′‐AAGAAGGTGGTGAAGCAGGCATC‐3′
	Reverse	5′‐CGGCATCGAAGGTGGAAGAGTG‐3′
*Abat*	Forward	5′‐CTTGCTGCTGGCTGAGGTCATC‐3′
	Reverse	5′‐GGAGTGTCGAAGGAACAGAAGGTG‐3′
*Atg5*	Forward	5′‐CAGAAGCTGTTCCGGCCTGTG‐3′
	Reverse	5′‐CAGATGCTCGCTCAGCCACTG‐3′
*Becn1*	Forward	5′‐GGACCAGGAGGAAGCTCAGTACC‐3′
	Reverse	5′‐CGCTGTGCCAGATGTGGAAGG‐3′
*Cad*	Forward	5′‐GCCAGCACATCCTGTCTGTCAAG‐3′
	Reverse	5′‐TCCGCTCCTTCTGTACCATCATCC‐3′
*Gad1*	Forward	5′‐AGTCTCTGGAGCAGATCCTGGTTG‐3′
	Reverse	5′‐TTGGTATTGGCAGTCGATGTCAGC‐3′
*Gfpt1*	Forward	5′‐CCAGAACGCTCTTCAGCAGGTG‐3′
	Reverse	5′‐CAACATCGTAGCCTCTCAGCACAG‐3′
*Gls*	Forward	5′‐GTCCTGAGGCAGTTCGGAATACAC‐3′
	Reverse	5′‐GAGGAGGAGACCAACACATCATGC‐3′
*Glud1*	Forward	5′‐GCATCTTGGAGGCTGACTGTGAC‐3′
	Reverse	5′‐TGGCACCTTCAGCAATGATCTTGG‐3′
*Glul*	Forward	5′‐CAAGAGGCACCAGTACCACATTCG‐3′
	Reverse	5′‐TTCTTCTCCTGGCCGACAGTCC‐3′
*Ppat*	Forward	5′‐GGTGCATCGCTTCTGGAGACTG‐3′
	Reverse	5′‐TCCTGACCTCGGTGCTGTAGC‐3′
*Map1lc3b*	Forward	5′‐CGTCCGAGAAGACCTTCAAGCAG‐3′
	Reverse	5′‐GTGGTCAGGCACCAGGAACTTG‐3′

### 2.18 Determination of glutamine content

PM cells of WT or *Hck* ^−/−^ mice, and PM cells of patients with or without EMs were collected to determine the concentration of glutamine by using Glutamine Colorimetric Assay Kit (BioVision, K556‐100), according to the manufacturer's instructions.

### 2.19 Gene microarray

The NC and HCKi THP‐1 cells were harvested and examined using the gene microarray Affymetrix GeneChip® Human Gene 2.0 ST Array (Affymetrix Inc.) as detailed in previous literature.^22^ The STRING database (accessible online at http://string-db.org) was utilised for prediction of Protein–Protein Interaction (PPI) networks.

### 2.20 Statistical analysis

Every experiment was conducted independently at least three times. For datasets comprising two groups, the Student's *t* test was utilised. In cases where the dataset consisted of more than two groups and followed a normal distribution, an analysis of variance (ANOVA) test was employed, followed by either Tukey or Bonferroni test for *t* tests. The results were expressed as mean ± standard error of the mean (SEM). All statistical analyses were performed using the SPSS 20.0 Statistical Package for the Social Sciences software. A significance level of *p* < 0.05 was considered to indicate a statistically significant difference.

## 3 RESULTS

### 3.1 The deficiency of HCK results in the impaired macrophage phagocytosis in EMs patients

**FIGURE 1:**
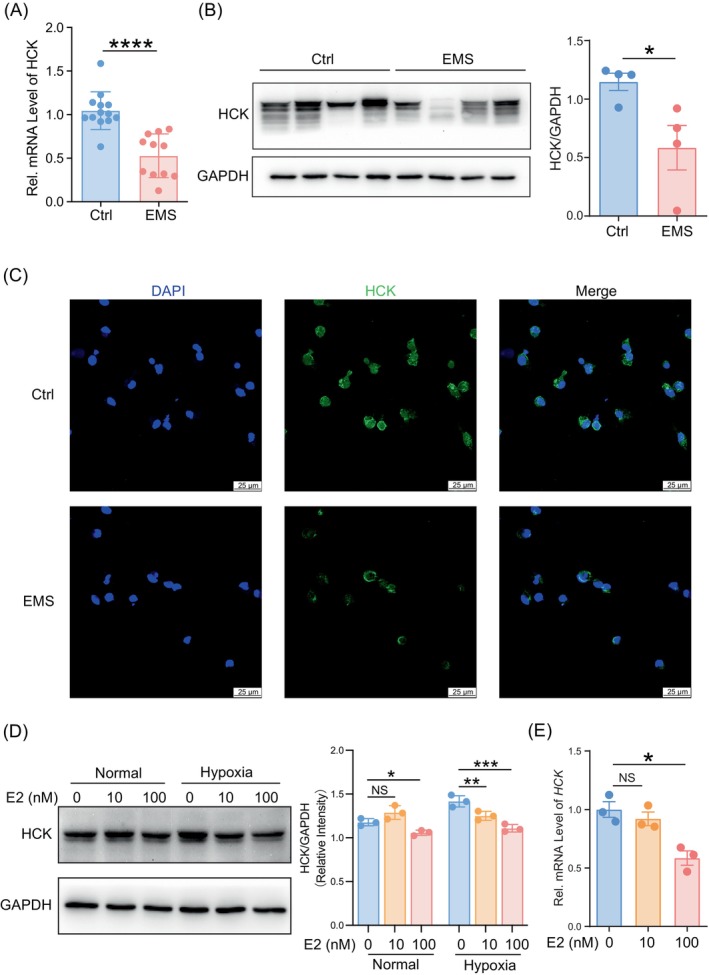
Hypoxia plus oestrogen decrease HCK levels of PM cells from EMs patients. (A) Transcription level of *HCK* in PM cells of patients of EMs group (*n* = 14) or Ctrl group (*n* = 11) by RT‐PCR. (B) Western‐blot for HCK expression of patients of EMs group (*n* = 4) or Ctrl group (*n* = 4). And quantitative analysis of the expression levels of HCK. (C) Immunofluorescence detection for HCK expression in PM cells of patients of EMs group (*n* = 4) or Ctrl group (*n* = 4) (Scale bar, 25 μm). (D) Western‐blot for HCK expression of M0 macrophages treated with estradiol (0 M, 10^−8 M, 10^−7 M) or estradiol combined with hypoxia (oxygen concentration settled at 2%) treatment for 48 h (*n* = 3) (left). The quantification of relative expression levels of HCK in M0 macrophages treated with estradiol (0 M, 10^−8 M, 10^−7 M) or estradiol combined with hypoxia (oxygen concentration setted at 2%) treatment for 48 h by normalisation with expression level of GAPDH (right). (E) Transcription level of *HCK* in PM cells of M0 macrophages treated with estradiol (0 M, 10^−8 M, 10^−7 M) for 24 h (*n* = 3). Data were presented as mean ± SEM and analysed by *t* test or one‐way ANOVA test. NS: no significance. *p < 0.05; **p < 0.01; ****p < 0.0001. EM, endometriosis; HCK, haematopoietic cellular kinase; PM, peritoneal macrophages; RT‐PCR, real‐time polymerase chain reaction.

To observe the expression of HCK in macrophages of PF, the PMs from control and EMs patients were isolated and purified. As shown, the expression of HCK in PF from EMs patients was notably lower in comparison to the control group (Figure 1A–C). EMs has always been considered as an oestrogen‐dependent disease,^29^ and hypoxia also plays an important role in origin and development of EMs.^30^ Here we observed exposure with low concentrations of estradiol did not influence the expression of HCK in PM in vitro, however, high concentration of estradiol with or without hypoxia condition obvious down‐regulated the HCK levels in PM (Figure 1D,E). These data suggest that endocrine and hypoxia environment lead to the downregulation of HCK in PM of EMs patients.

**FIGURE 2:**
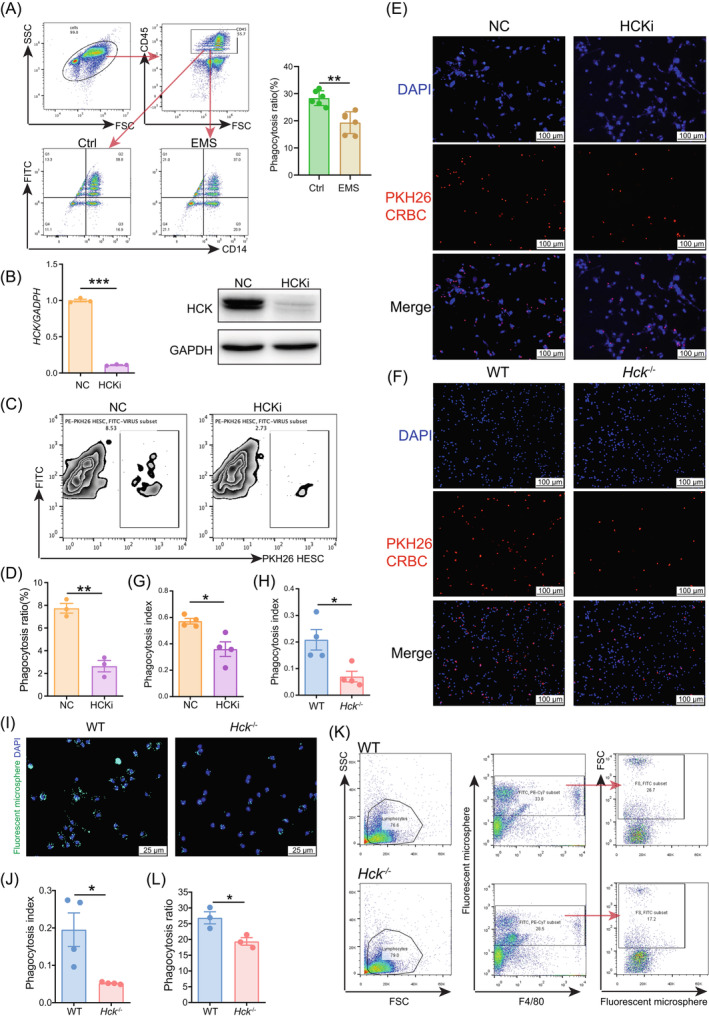
Deficiency of HCK leads to insufficient phagocytosis of macrophages. (A) The PM cells of patients with or without EMs were incubated with carboxylate‐modified fluorescent latex beads for 1 h (number of macrophages: number of beads = 1:100). Phagocytosis ratio was detected by FCM and calculated as the percentage of CD14^+^FITC^+^ cells among CD14^+^ macrophages. (B) Validation of *HCK* silenced THP1 cell lines using Western blotting or RT‐PCR. (C, D) HESCs were labelled with PKH26 and co‐cultured with NC (*n* = 3) or HCKi (*n* = 3) M2 macrophages for 2 h (number of macrophages: number of HESCs = 1:2). Phagocytosis ratio was detected by FCM and calculated as the percentage of PKH26^+^ macrophages among total macrophages. (E–H) Chicken erythrocytes (CRBC) were labelled with PKH26 and co‐cultured with NC (*n* = 3) or HCKi (*n* = 3) M2 macrophages (E and G) or PM cells of *Hck* ^−/−^ mice (*n* = 4) or WT mice (*n* = 4) (F and H) for 1 h (number of macrophages: number of chicken erythrocytes = 1:10). Macrophages labelled with DAPI. Phagocytosis was detected by fluorescence microscopy. The phagocytic index calculated as the percentage of phagocyted chicken erythrocytes among total macrophages (Scale bar, 100 μm). (I–L) The PM cells of *Hck* ^−/−^ mice (*n* = 4) or WT mice (*n* = 4) were incubated with carboxylate‐modified fluorescent latex beads for 1 h (number of macrophages: number of beads = 1:100). Phagocytosis was detected by fluorescence microscopy (I, J) or FCM (K, L) (Scale bar, 25 μm). Data were presented as mean ± SEM and analysed by *t* test. *p < 0.05; **p < 0.01; ***p < 0.001. EM, endometriosis; FCM, flow cytometry; HCK, haematopoietic cellular kinase; HCKi, HCK‐silenced; HESC, human endometrial stromal cells line; NC, normal control THP‐1 cells; PM, peritoneal macrophages.

Consistent with previous reports,^10^, ^11^ the impaired PM phagocytosis to carboxylate‐modified fluorescent latex beads was observed in EMs patients (Figure 2A). To further clarify the possible role of HCK in PM phagocytosis, we analysed the phagocyte ability of control THP‐1 cells and HCK‐silenced (HCKi) M2 macrophages (Figure 2B) against PKH26‐labled human ESC line (hESC). As shown, silencing HCK markedly decreased macrophage phagocytosis to hESC (Figure 2C,D). Similarly, there were impaired phagocyte abilities of HCKi M2 macrophages and peritoneal macrophages from *Hck* ^−/−^ mice to PKH26‐labled chicken erythrocyte (Figure 2E–H) or fluorescent latex beads (Figure 2I–L) in vitro. Therefore, these data suggest that the abnormal decrease in HCK levels may play a role in the compromised PM phagocytosis in EMs.

### 3.2 Macrophage phagocytosis induced by the HCK is dependent on glutamine level

**FIGURE 3:**
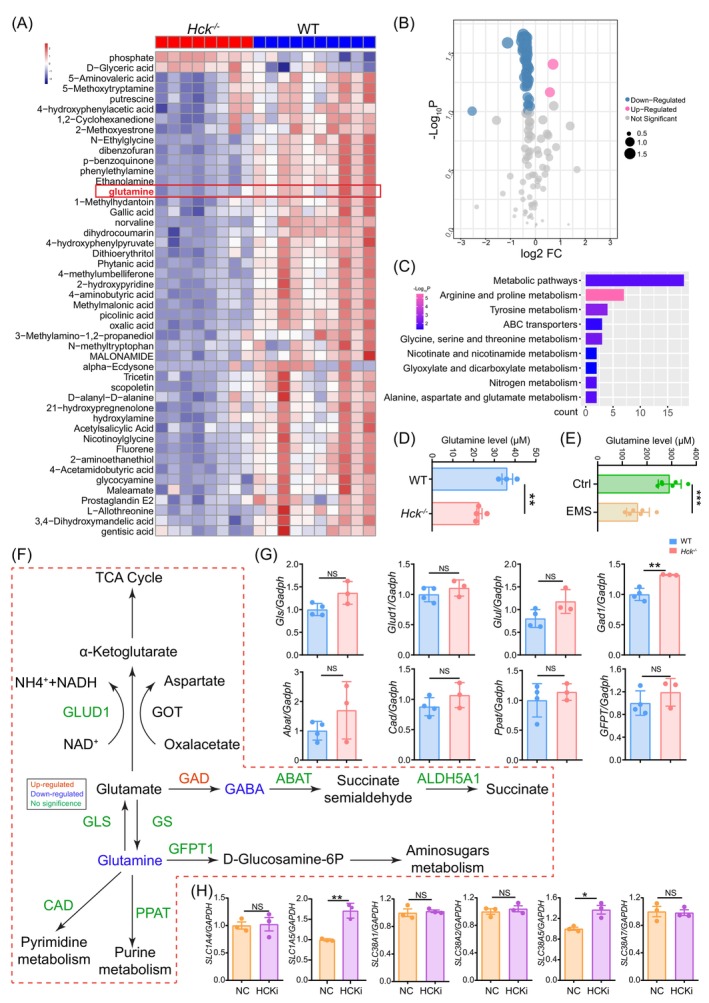
EMs‐induced reduction of HCK leads to decreased glutamine levels. (A) Heatmap of differential metabolites in PM cells of *Hck* ^−/−^ mice (*n* = 8) and WT mice (*n* = 10). (B) Volcano plot showing differential metabolites of PM cells of *Hck* ^−/−^ mice (*n* = 8) or WT mice (*n* = 10). Red colour indicates metabolites that are upregulated and blue colour indicates downregulated metabolites in *Hck* ^−/−^ mice when compared with the WT mice. (C) Enriched KEGG pathways of differential metabolites clustering form (A). (D) Glutamine levels of PM cells of *Hck* ^−/−^ mice (*n* = 4) or WT mice (*n* = 3) were determined. (E) Glutamine levels of PM cells of patients of EMs group (*n* = 6) or Ctrl group (*n* = 6) were determined. (F) Summary of glutamine metabolism characteristics in PM cells of *Hck* ^−/−^ mice (ABAT: aminobutyrate aminotransferase, ALDH5A1: aldehyde dehydrogenase 5 family, member A1, CAD: carbamoyl phosphate synthase, GABA: γ‐aminobutyrate, GAD: glutamate decarboxylase, GFPT1: glutamine‐fructose‐6‐phosphate transaminase 1, GLS: glutaminase, GLUD1:glutamate dehydrogenase1, GS: glutamine synthetase, GOT: glutamic oxaloacetic transaminase, PPAT: phosphoribosyl pyrophosphate amidotransferase, TCA Cycly: tricarboxylic acid cycle). (G) Expression of metabolic enzymes of glutamine metabolism between PM cells of *Hck* ^−/−^ mice (*n* = 3) or WT mice (*n* = 4) was detected by RT‐PCR. (H) Expression of glutamine transporters of NC (*n* = 3) or HCKi (*n* = 3) THP‐1 cells was detected by RT‐PCR. Data were presented as mean ± SEM or median and quartile and analysed by *t* test. *p < 0.05; **p < 0.01; ***p < 0.001. EM, endometriosis; HCK, haematopoietic cellular kinase; HCKi, HCK‐silenced; NC: normal control THP‐1 cells; NS: no significance; PM, peritoneal macrophages.

In order to examine the potential metabolic process responsible for the lack of HCK resulting the impaired PM phagocytosis, a metabolomics analysis was recruited to evaluate the differential metabolites of PM cells between wide type (WT, *n* = 8) and *Hck* ^−/−^ (*n* = 10) mice. As shown in Figure 3A, we found two up‐regulated metabolites and 45 down‐regulated metabolites in PM from *Hck* ^−/−^ mice, there are mainly enriched in the arginine and proline metabolism, alanine, aspartate and glutamate metabolism (Figure 3B,C). Further analysis showed that glutamine level in PM of *Hck* ^−/−^ mice and EMs patients was decreased (Figure 3D,E), and the level of glutamate decarboxylase 1 (GAD1), a downstream metabolic enzyme of glutamine, was increased (Figure 3F,G). However, the expression of glutamine synthetase (glutamine synthetase [GS]) and catabolic enzyme (glutaminase [GLS], glutamine‐fructose‐6‐phosphate transaminase 1 [GFPT1], carbamoyl phosphate synthase [CAD] and phosphoribosyl pyrophosphate amidotransferase [PPAT]) were unchanged (Figure 3F,G). Of note, glutamine transporters, including SLC1A5 and SLC38A5 were up‐regulated in HCKi THP‐1 cells (Figure 3H), which mediate glutamine uptake.^31^ This may be a compensatory response caused by a decrease in macrophage glutamine levels.

**FIGURE 4:**
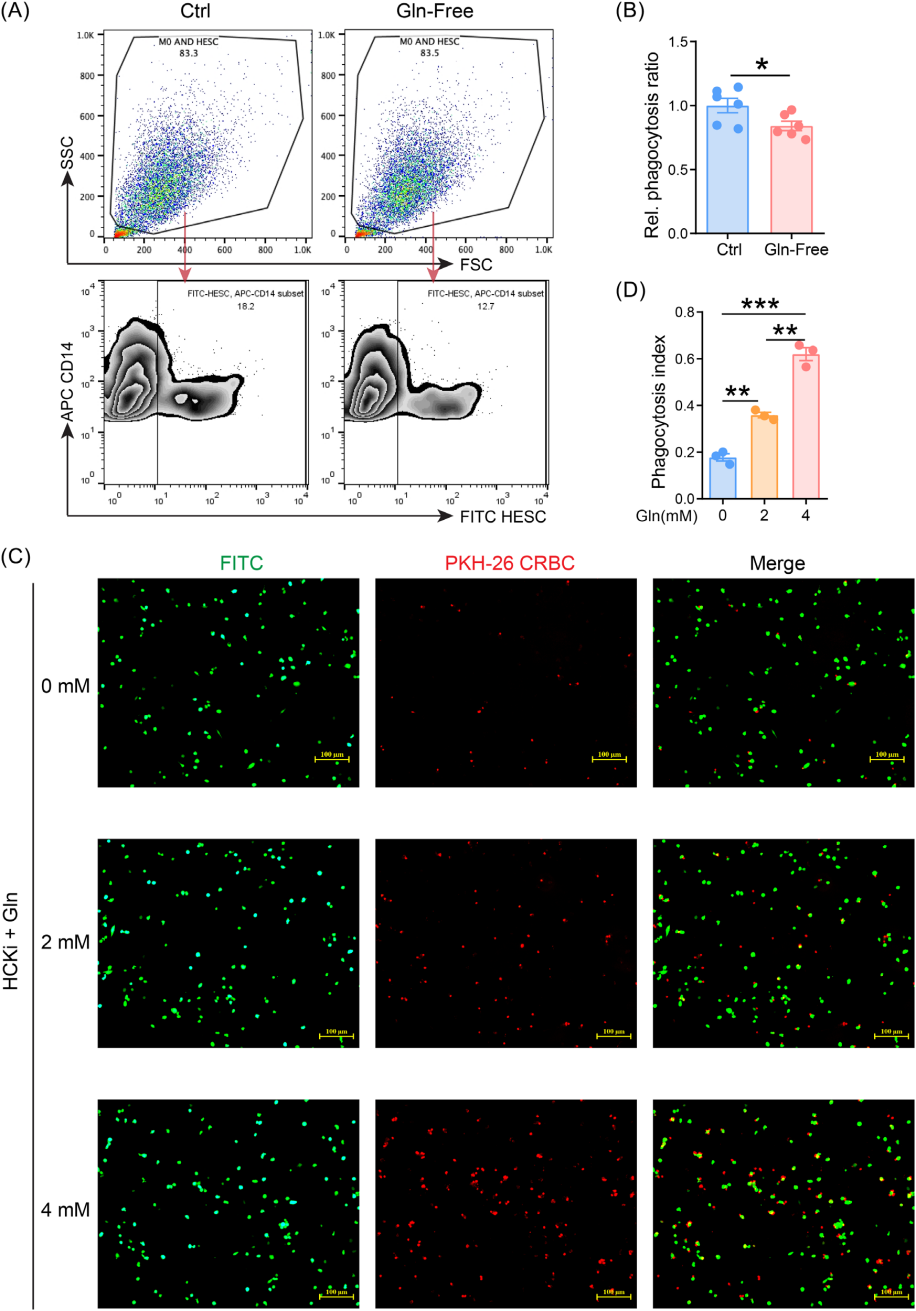
Glutamine enhances phagocytosis of HCKi macrophages. (A, B) HESC that were labelled with CFSE were co‐cultured with M2 macrophages in either glutamine‐free (*n* = 6) or control medium (*n* = 6) for a duration of 2 h (number of macrophages: number of HESCs = 1:2). Phagocytosis was detected by FCM and calculated as the percentage of FITC^+^APC^+^ cells among APC^+^ macrophages. (C, D) HCKi M2 macrophages treated with glutamine (0 mM, 2 mM, 4 mM) for 4 h. After that, chicken erythrocytes (CRBC) that were labelled with PKH26 were co‐cultured with HCKi macrophages pre‐treated with glutamine for a duration of 1 h (number of macrophages: number of chicken erythrocytes = 1:10). Phagocytosis was detected by fluorescence microscopy (Scale bar, 100 μm). Data were presented as mean ± SEM and analysed by *t* test or one‐way ANOVA test. *p < 0.05; **p < 0.01; ***p < 0.001. HCK, haematopoietic cellular kinase; HCKi, HCK‐silenced.

In order to investigate the potential impact of glutamine on macrophage phagocytosis, THP‐1 cells were induced to differentiate to M2 macrophages, and then co‐cultured with CSFE‐labelled ESCs in glutamine deprivation (Gln‐free) or control medium for 2 h. As depicted in Figure 4A,B, compared to control medium, Gln‐free led to the decreased phagocytosis of macrophages to ESCs. In contrast, glutamine enhanced the phagocyte ability of HCKi M2 macrophages to chicken erythrocyte in a dosage‐dependent manner (Figure 4C,D), suggesting that the decreased glutamine level induced by HCK deficiency should contribute to the macrophage phagocytosis dysfunction in EMs.

### 3.3 Glutamine suppresses macrophage autophagy

**FIGURE 5:**
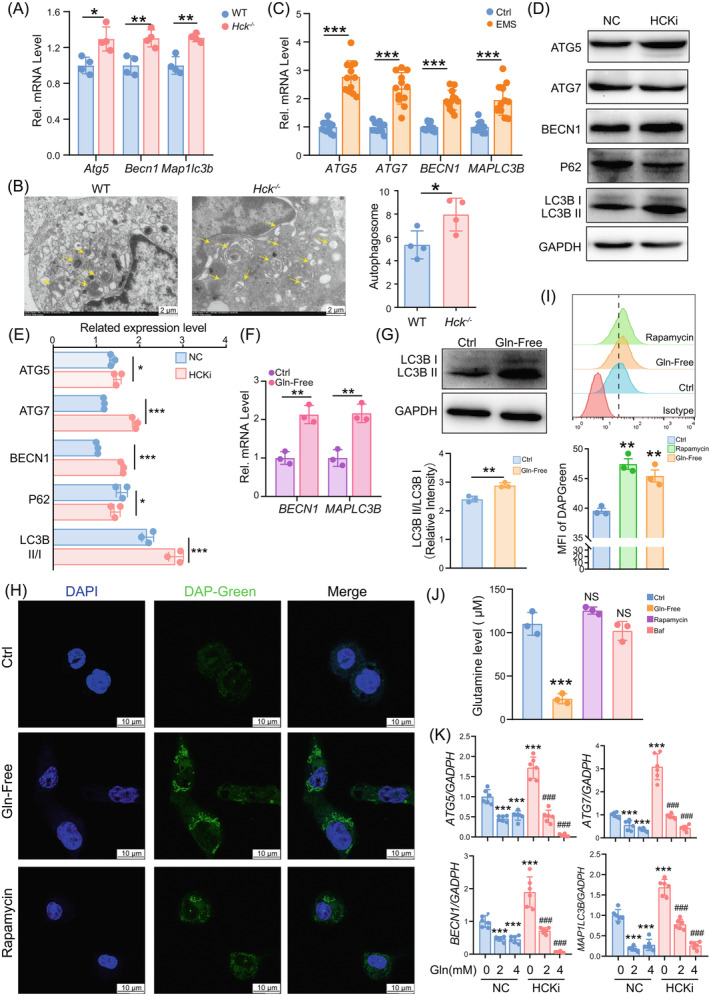
A dysfunctional HCK/glutamine pathway induces upregulation of autophagy in macrophages. (A) Transcription level of autophagy related genes in PM cells of *Hck* ^−/−^ mice (*n* = 4) or WT mice (*n* = 4) by RT‐PCR. (B) Autophagic structures (indicated by yellow arrows) under transmission electron microscopy in PM cells of *Hck* ^−/−^ mice (*n* = 4) or WT mice (*n* = 4) (Scale bar, 10 μm). (C) Transcription level of autophagy related genes in PM cells of EMs group (*n* = 12) or Ctrl group (*n* = 10) by RT‐PCR. (D) Expression of autophagy related proteins in NC or HCKi macrophages was detected by Western blotting. (E) The quantification of relative protein expression levels in (D) in NC or HCKi macrophages by normalisation with expression level of GAPDH. (F, G) Transcription level of autophagy related genes in M2 macrophages that were cultured in either glutamine‐free (*n* = 3) or regular growth medium (*n* = 3) for a duration of 4 h by RT‐PCR (F) and Western blotting (G). (H, I) Immunofluorescence detection (H) or FCM analysis (I) for autophagy detection of M2 macrophages treated with regular growth medium, glutamine‐free medium, or rapamycin (100 nM) (*n* = 3) for 4 h (Scale bar, 10 μm). (J) Glutamine levels of M2 macrophages pre‐treated with regular growth medium, glutamine‐free medium, rapamycin (100 nM) for 4 h, or bafilomycin a1 (Baf, 100 μM) (*n* = 3) for 1 h were determined. (K) Transcription level of autophagy related genes in NC or HCKi macrophages (cultured with glutamine‐free medium) treated by glutamine 0 mM, 2 mM, 4 mM for 4 h, by RT‐PCR. Data were presented as mean ± SEM and analysed by *t* test or one‐way ANOVA test. *p < 0.05; **p < 0.01; ***p < 0.001; ^###^ *p* < 0.001. EM, endometriosis; FCM, flow cytometry; HCK, haematopoietic cellular kinase; HCKi, HCK‐silenced; NC: normal control THP‐1 cells; PM, peritoneal macrophages; RT‐PCR, real‐time polymerase chain reaction.

Glutamine plays a role in controlling cell autophagy, which is a key factor for macrophage differentiation and function.^32^, ^33^, ^34^ Further analysis indicated that autophagy‐related molecules (*Atg5*, *Becn1*, *Map1lc3b*) expression and autophagosome number were significantly up‐regulated in PM of *Hck* ^−/−^ mice (Figure 5A,B). In human, there were also decreased expressions of ATG5, ATG7, BECN1 and MAPLC3B in PF from EMs patients, and silencing HCK led to the up‐regulation of ATG5, BECN1 and LC3B, and the down‐regulation of P62 in THP1 cells (Figure 5C–E). More importantly, Gln‐free resulted in the increase of autophagy of macrophages in vitro (Figure 5F–I), suggesting that glutamine restricts macrophage autophagy. However, either rapamycin (an autophagy inducer) or Bafilomycin A1 (Baf) (an autophagy inhibitor) did not influence the level of glutamine in macrophages (Figure 5J). More interestingly, the stimulatory effect of silencing HCK on the levels of autophagy‐related molecules (ATG5, ATG7, BECN1 and MAP1LC3B) was markedly inhibited by glutamine (Figure 5K). These findings illiterate that macrophage phagocytosis activated by glutamine is dependent on the suppression of cell autophagy.

### 3.4 Glutamine maintains macrophage phagocytosis by suppressing the c‐FOS/c‐JUN signal pathway‐mediated autophagy

**FIGURE 6:**
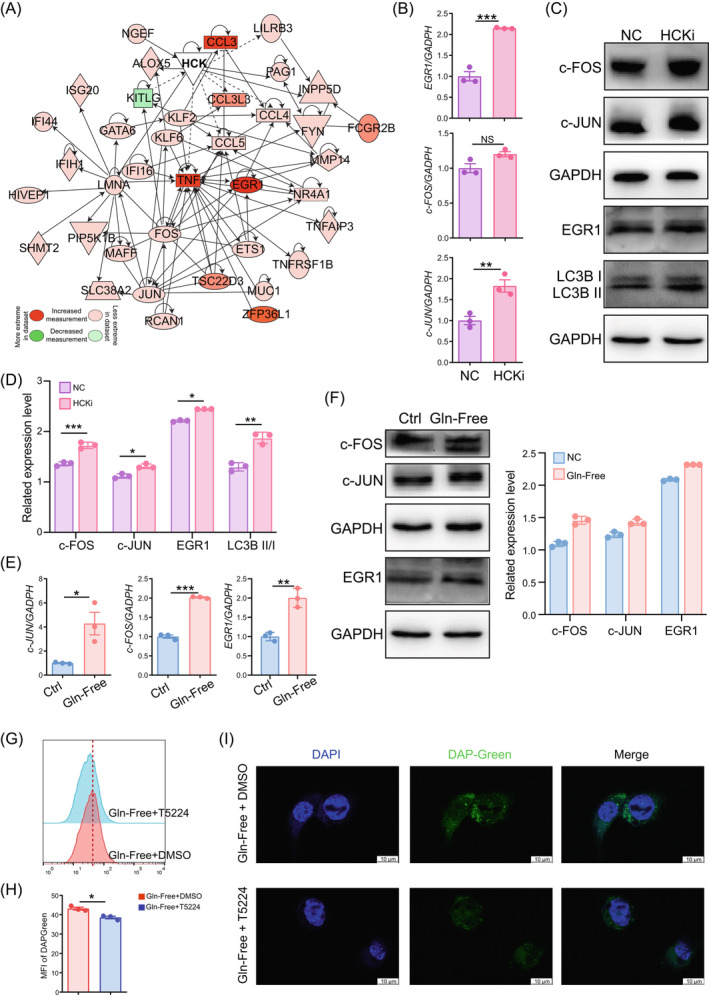
Macrophage autophagy induced by lack of HCK/glutamine is dependent on the activation of c‐FOS/JUN. (A) The PPI of differentially expression genes between NC and HCKi macrophages (*n* = 3). (B) Transcription level of essential genes in (A) of NC and HCKi macrophages by RT‐PCR. (*n* = 3). (C) Expressions of essential proteins or autophagy related proteins in NC or HCKi macrophages were detected by Western blotting. (D) The quantification of relative protein expression levels in (C) in NC or HCKi macrophages by normalisation with expression level of GAPDH. (E) Transcription level of essential genes in (A) of M2 macrophages in either glutamine‐free (*n* = 3) or regular growth medium (*n* = 3) for a duration of 4 h. (F) Expressions of essential proteins in M2 macrophages in either glutamine‐free or regular growth medium for a duration of 4 h were detected by Western blotting (left). The quantification of relative protein expression levels in M2 macrophages in either glutamine‐free or regular growth medium for a duration of 4 h by normalisation with expression level of GAPDH (right). (G–I) FCM analysis (G, H) or immunofluorescence detection (I) for autophagy detection of M2 macrophages cultured with glutamine‐free medium with/without T5224 (10 μM) (*n* = 3) for 4 h (Scale bar, 10 μm). Data were presented as mean ± SEM and analysed by *t* test. *p < 0.05; **p < 0.01; ***p < 0.001, NS: no significance. HCK, haematopoietic cellular kinase; HCKi, HCK‐silenced; NC: normal control THP‐1 cells; PPI, protein–protein interaction; RT‐PCR, real‐time polymerase chain reaction.

To analyse the potential mechanisms of HCK and glutamine in macrophage autophagy, RNA‐sequencing was applied, as well as the PPI Networks analysis of differential expressed genes between ctrl and HCKi THP‐1 cells (Figure 6A). The data of western blotting and qPCR showed that silencing HCK led to the increases of c‐FOS, c‐JUN and or LC3B in THP‐1 cells (Figure 6B–D). Similarly, exposure to Gln‐free resulted in the up‐regulation of c‐FOS and c‐JUN levels (Figure 6E,F). More importantly, the autophagy levels induced by Gln‐free was partially inhibited by T5224 (an inhibitor for the DNA binding activity of c‐Fos/c‐Jun) in THP‐1 cells (Figure 6G–I), indicating that glutamine inhibits macrophage autophagy partially via the c‐FOS/c‐JUN signal pathway.

**FIGURE 7:**
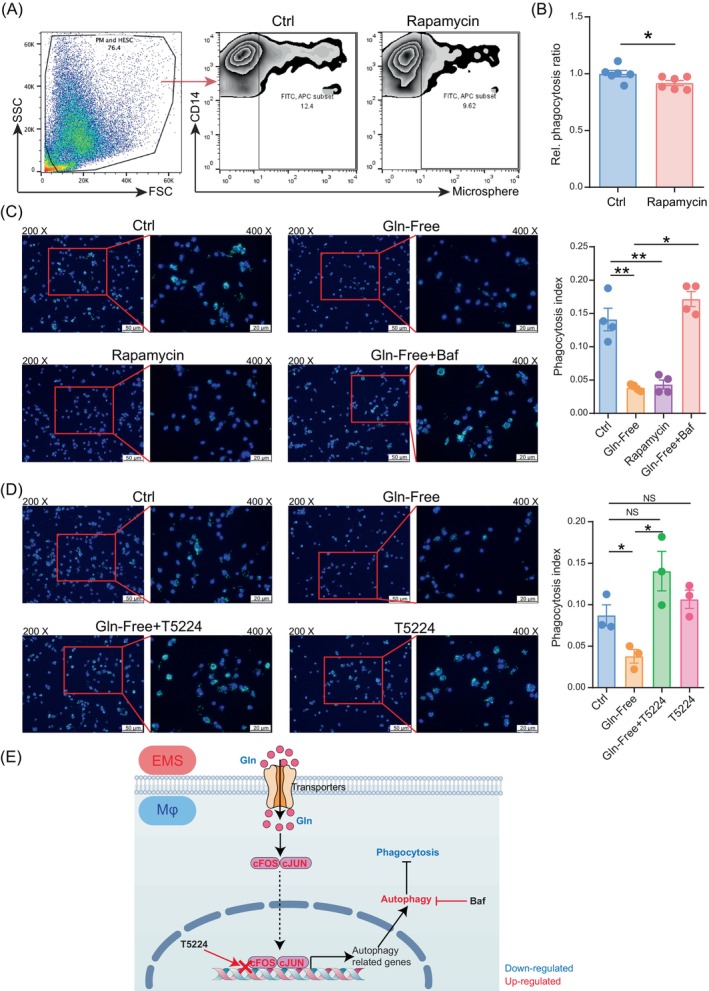
Autophagy restricts macrophage phagocytosis. (A, B) M2 macrophages pre‐treated with regular growth medium or rapamycin (100 nM) for 4 h, and then M2 macrophages were incubated with carboxylate‐modified fluorescent latex beads for 1 h (number of macrophages: number of beads = 1:100). Phagocytosis was detected by FCM and calculated as the percentage of CD14^+^FITC^+^ cells among CD14^+^ macrophages (*n* = 6). (C) M2 macrophages pre‐treated with regular growth medium, glutamine‐free medium, rapamycin (100 nM) for 4 h, or bafilomycin a1 (Baf, 100 μM) combine with glutamine‐free medium for 1 h, were incubated with carboxylate‐modified fluorescent latex beads for 1 h (number of macrophages: number of beads = 1:100). Phagocytosis was detected by fluorescence microscopy (Scale bar, 50 μm or 20 μm). (D) M2 macrophages pre‐treated with regular growth medium, glutamine‐free medium, T5224 (10 μM) or T5224 (10 μM) combine with glutamine‐free medium for 4 h, were incubated with carboxylate‐modified fluorescent latex beads for 1 h (number of macrophages: number of beads = 1:100). Phagocytosis was detected by fluorescence microscopy (Scale bar, 50 μm or 20 μm). (E) Schematic diagram of the regulation of glutamine/FOS/JUN‐autophagy‐phagocytosis axis. In the microenvironment of endometriosis, the reduced glutamine level of peritoneal macrophages results in elevated cFOS and cJUN expression. This leads to upregulation of autophagy‐related genes, thereby suppressing macrophage phagocytic function. Restoring macrophage phagocytic ability can be achieved by inhibiting macrophage autophagy (Baf treatment) or by blocking the DNA binding capacity of cFOS/cJUN (T5224 treatment). Data were presented as mean ± SEM and analysed by *t* test or one‐way ANOVA test. *p < 0.05; **p < 0.01; ***p < 0.001. NS: no significance.

Subsequently, the roles of autophagy and the c‐FOS/c‐JUN signal pathway in macrophage phagocytosis were evaluated in vitro. We noticed that both Gln‐free and rapamycin enhanced macrophage phagocytosis against carboxylate‐modified fluorescent latex beads (Figure 7A–C). Interestingly, both Baf and T5224 could reverse the inhibitory effect of Gln‐free on macrophage phagocytosis (Figure 7C,D). These data indicate that glutamine inhibits autophagy and further macrophage phagocytosis by suppressing the c‐FOS/c‐JUN axis (Figure 7E).

### 3.5 HCK deficiency impairs macrophage phagocytosis and accelerates the progress of EMs by the glutamine‐autophagy axis

**FIGURE 8:**
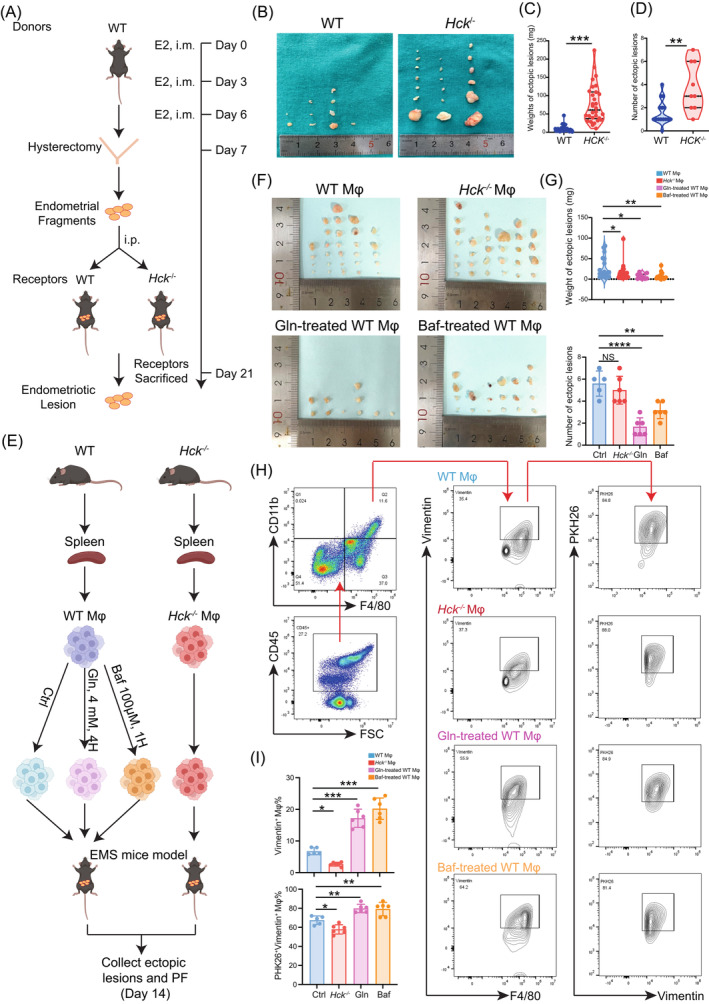
Glutamine suppresses endometriosis development by regulating autophagy and phagocytosis of macrophage. (A) This flowchart depicts the steps involved in establishing a mouse model for allogeneic endometrial transplantation using intraperitoneal injection of fragments of endometrial tissue. (B–D) The pictures (B), weight (C) and number (D) of ectopic lesions were analysed. (E) The process of constructing an in vivo phagocytosis model. (F, G) The pictures (F), weight and number (G) of ectopic lesions were analysed. (H, I) The proportions of Vimentin^+^ and PKH26^+^ macrophages were evaluated by FCM. Phagocytosis was calculated as the percentage of Vimentin^+^PKH26^+^ macrophages cells among donor macrophages. Data were presented as mean ± SEM and analysed by *t* test or one‐way ANOVA test. *p < 0.05; **p < 0.01; ***p < 0.001; ****p < 0.0001. FCM, FCM, flow cytometry; NS: no significance.

In *Hck* ^−/−^ mice, we observed that both the weight and number of ectopic lesions were greater compared to those in WT mice (Figure 8A–D). To further explore the potential role of the HCK‐glutamine‐autophagy axis on macrophage phagocytosis and disease development, we collected WT macrophage, *Hck* ^−/−^ macrophage, glutamine‐pre‐treated macrophage and Baf‐pre‐treated macrophage, labelled these cells with PKH26, and intraperitoneally into EMs mice (Figure 8E). As shown, there were the increased weight of ectopic lesion, and decreased phagocyte ability of macrophage against vimentin^+^ ESC in the *Hck* ^−/−^ macrophage transfer group (Figure 8F–I). In contrast, glutamine‐pre‐treated macrophage and Baf‐pre‐treated macrophage led to the reduced weight and number of ectopic lesion and the enhanced macrophage phagocytosis in EMs mice (Figure 8F–I). Based on these data, it can be inferred that the low level of HCK impairs macrophage phagocytosis and accelerates the progress of EMs, and these effects should be dependent on the glutamine‐autophagy axis.

## 4 DISCUSSION

Macrophage dysfunction is involved in the establishment and development of EMs by multiple pathways, including inflammation, promotion of endometrial cell proliferation and invasion, angiogenesis, attenuated phagocytosis and so forth.^35^ The impaired macrophage phagocytosis ultimately leads to the clearance deficiencies of ectopic endometrial cells, however, the mechanism for the impaired macrophage phagocytosis is largely unknown. Here we found the expression of HCK in PM from EMs patients was decreased, as well as the phagocyte ability. Interestingly, exposure to oestrogen plus hypoxia condition results in the decreased of HCK in macrophage. According to previous research, STAT3 signalling is engaged in the regulation of HCK expression, under the negative regulation of oestrogen.^22^ Additionally, STAT3 and hypoxia inducible factor‐1α (HIF1α) mediate the transcriptional and physiological responses to hypoxia.^36^ Lactate has been reported to be a pivotal factor that drive M2 macrophage polarisation by the activation of ERK/STAT3 signalling pathway in EMs.^37^ Therefore, hypoxia and oestrogen cooperatively decrease HCK in macrophage, possibly via the STAT3 signalling pathway, which warrants further research.

Metabolism reprogramming plays important roles in macrophage phagocytosis, including glycolysis and heme.^14^, ^15^, ^18^ Glutamine is the most abundant nonessential amino acid in the human body. Moreover, recent evidence has demonstrated that glutamine promotes M2 macrophage polarisation through the α‐ketoglutarate and glutamine–UDP‐GlcNAc pathways.^38^ More importantly, we observed there was low levels of glutamine in macrophage of Hck−/− mice and EMs patients. Further analysis suggested that HCK deficiency contributes to the macrophage phagocytosis dysfunction in EMs partly by decreasing glutamine level. Glutamine deprivation has been reported to induce PD‐L1 expression via the activation of EGFR/ERK/c‐Jun signalling in cancer cells.^39^, ^40^ In addition, the JNK/c‐Jun signalling pathway is involved in the regulation of cell autophagy.^41^, ^42^ Here, we found that insufficient glutamine led to the enhanced autophagy and impaired phagocytosis of macrophage, and this effect is also dependent on the c‐FOS/c‐JUN signal pathway. Owing to the key role of glutamine metabolism‐AMPK‐MTORC1 signalling axis in autophagy regulation,^32^ it is feasible to speculate that this regulatory axis may be also involved in the autophagy regulation in macrophages.

In mice model, *Hck* ^−/−^ macrophage increased weight of ectopic lesion, and decreased phagocyte ability of macrophage. More differentially, glutamine‐pre‐treated macrophage and Baf‐pre‐treated macrophage led to the decreased weight and number of ectopic lesion and the increased macrophage phagocytosis in EMs mice, indicating that the aberrant HCK‐glutamine‐autophagy axis impairs macrophage phagocytosis and accelerates the progress of EMs. For removing and recycling intracellular elements, basal autophagy is critical to maintain macrophage function, including survival and phagocytosis. In current study, *Hck* ^−/−^ and insufficient glutamine resulted in the increases of LC3B and autophagy of macrophage with phagocytosis dysfunction. Literatures suggested that autophagy, ATG7 and LC3 participate in the induction of cell phagocytosis.^43^, ^44^, ^45^, ^46^ The relationship between phagocytosis and autophagy is more complex, and ROS signal transduction might be involved in both processes.^47^, ^48^ However, the exact mechanism of autophagy in macrophage phagocytosis in EMs still needs further exploration.

**FIGURE 9:**
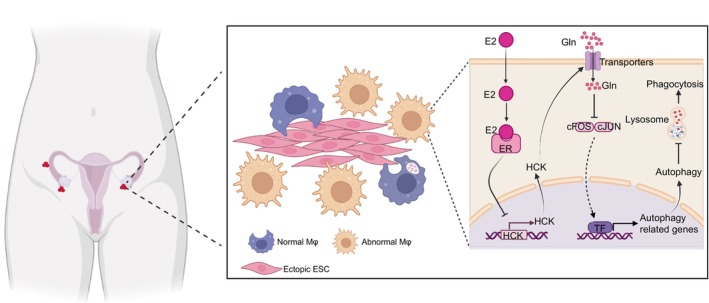
Schematic roles of the inhibition of phagocytic function in PM cells as a result of low HCK expression in patients with EMs. Abnormal elevation of oestrogen levels in patients with EMs leads to a reduction in HCK expression in PM cells, which subsequently affects glutamine transport, resulting in reduced intracellular glutamine content. Low glutamine levels in macrophages cause an increase in expression of EGR1, cFOS and cJUN, promoting transcription of autophagy‐related genes, further leading to a decrease in macrophage phagosomes and impaired phagocytic function. Ultimately, this results in insufficient clearance of endometrial cells at ectopic sites and the formation of endometriotic foci. EM, endometriosis; HCK, haematopoietic cellular kinase; PM, peritoneal macrophages.

Collectively, as displayed in Figure 9, exposure to high level of oestrogen, accompanied by the hypoxia condition, should decrease the expression of HCK in macrophage. HCK deficiency induces excessive autophagy of macrophage in a glutamine metabolism‐c‐FOS/c‐JUN signalling pathway, contributing to the impaired macrophage phagocytosis and EMs development. Results from this study shed light on immune metabolism and autophagy mechanism of macrophage phagocytosis and EMs. Excitingly, supplement with glutamine‐pre‐treated macrophage or macrophage with low autophagy can enhance macrophage phagocytosis and suppress disease progression. Therefore, the potential therapeutic values of macrophage treated with glutamine or autophagy inhibitor in EMs should be emphasised due to the activation of macrophage phagocytosis in endometriotic milieu. However, the efficacy and safety also need further research.

## AUTHOR CONTRIBUTIONS

Both LST and LZZ made an equal contribution to this project. LST and LZZ performed the experiments, analysed data. LST, LZZ and HSH performed the animal experiments. LYK helped the collection of peritoneal fluid from EMS patients and controls. LMQ designed the study and wrote the manuscript. ZD supervised the work and revised the manuscript. All authors read and approved the final manuscript.

## FUNDING INFORMATION

This study was supported by the Major Research Program of National Natural Science Foundation of China (NSFC) (No. 81771548, 82071615, 92057119, 31970798); the Program for Zhuoxue of Fudan University (JIF157602) and the Support Project for Original Personalized Research of Fudan University (IDF157014/002).

## CONFLICT OF INTEREST STATEMENT

The authors declare that they have no competing interests.

## Data Availability

The data that support the findings of this study are available from the corresponding author upon reasonable request.
